# Ovarian juvenile granulosa cell tumors with Ollier’s disease in children with IDH1 gene somatic mutation

**DOI:** 10.3389/fendo.2023.1093273

**Published:** 2023-05-30

**Authors:** Jin Zhang, Renwu Hua, Lishuang Ma, Chao Liu, Yanxia Zhang, Xuemin Lü, Tianren Wang, Naijun Wan

**Affiliations:** ^1^ Department of Pediatrics, Beijing Jishuitan Hospital, Beijing, China; ^2^ Shenzhen Key Laboratory of Fertility Regulation, Reproductive Medicine Center, The University of Hong Kong-Shenzhen, Shenzhen, China; ^3^ Department of General Surgery, Capital Institute of Pediatrics, Beijing, China; ^4^ Department of Pediatric Orthopedics, Beijing Jishuitan Hospital, Beijing, China

**Keywords:** children, juvenile granulosa cell tumors, ovarian, Ollier’s disease, IDH1, gene

## Abstract

**Objective:**

The aim of this study was to explore the symptoms, treatment, and pathogenesis of ovarian juvenile granulosa cell tumors with Ollier’s disease in children.

**Methods:**

From October 2019 to October 2020, clinical data were retrospectively analyzed for one case of ovarian juvenile granulosa cell tumors with Ollier’s disease. Whole-exome sequencing and Sanger sequencing were used to detect gene mutation in ovarian tumor and chondroma tissue. NADP-dependent isocitrate dehydrogenase-1 (IDH1) and S6 ribosomal protein expression levels in cells transfected with wild-type or mutant plasmid were analyzed by Western blot.

**Results:**

The 4-year-old female showed multiple skeletal deformities, bilateral breast development with chromatosis, and vulvar discharge. Sex hormone assay suggested that estradiol and prolactin were elevated, and the x-ray of limbs suggested enchondroma. Pelvic ultrasound and abdominal CT revealed a right ovarian solid mass. Pathologic examination of the right ovarian solid mass showed a juvenile granulosa cell type. A c.394C>T (p. Arg132Cys) mutation of the IDH1 gene was detected in both the ovarian juvenile granulosa cell tumors and enchondroma. Transfection of HeLa cells with either WT or Mut plasmid caused 4.46- or 3.77-fold overexpression of IDH1 gene compared to non-transfected control cells, respectively. R132C mutation inhibited the phosphorylation of S6 ribosomal protein, which is central to the mTOR pathway. Postoperatively, estradiol and prolactin levels fell to values normal for her age and bilateral breast gradual retraction.

**Conclusion:**

The incidence of ovarian juvenile granulosa cell tumors with Ollier’s disease in children may be caused by generalized mesodermal dysplasia; IDH1 gene mutation may play a facilitated role in this process. Surgical operation is the main treatment. We suggest that patients with ovarian juvenile granulosa cell tumors and Ollier’s disease should undergo regular investigation.

## 1 Introduction

Ovarian granulosa cell tumor is an ovarian sex cord–stromal tumor with granulosa cell morphological characteristics and endocrine function, and it accounts for 2%–5% of all ovarian malignancies (1). There are two histologically and clinically distinct subtypes: adult granulosa cell tumors and juvenile granulosa cell tumors (JGCTs). The adult subtype accounts for 95% of granulosa cell tumors; the juvenile subtype accounts for 5% and characteristically develops before puberty. Multiple enchondromas are rare non-hereditary benign tumors first reported by Ollier in 1899, also known as Ollier’s disease (OD), with a prevalence of approximately 1/100,000 (2). At present, more than 10 cases of ovarian granulosa cell tumor combined with enchondroma have been reported. However, the pathogenesis between the two diseases is unknown. Here, we present a case of juvenile ovarian granulosa cell tumor accompanied by OD in a 4-year-old female. In order to further explore the possible associated pathogenesis between them, whole-exome sequencing and Sanger sequencing were used to detect gene mutation in the ovarian JGCT and chondroma tissue. In addition, the mutant gene was verified by cell experiment *in vitro*.

## 2 Materials and methods

### 2.1 Patient

**Figure 1:**
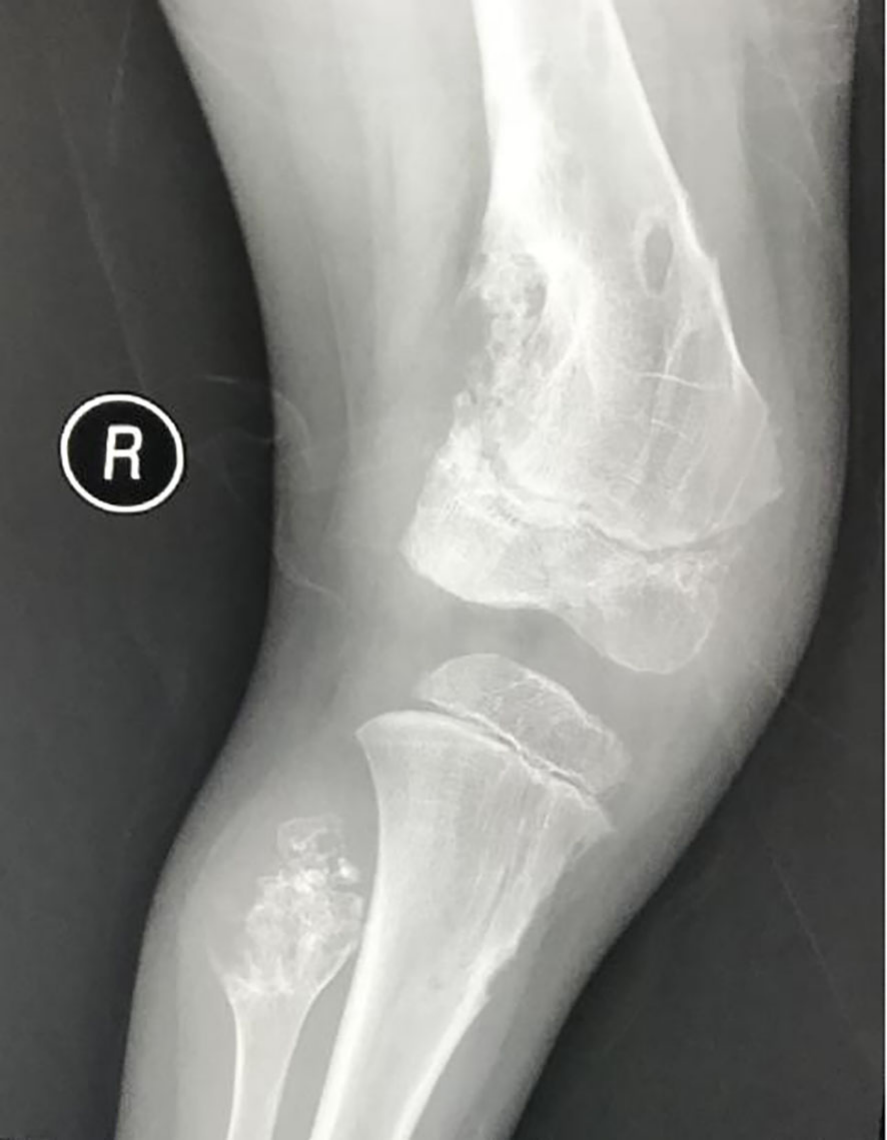
Plain bone X-ray showed multiple lytic bone lesions in distal part of the right femur, right tibia and fibula.

**Figure 2:**
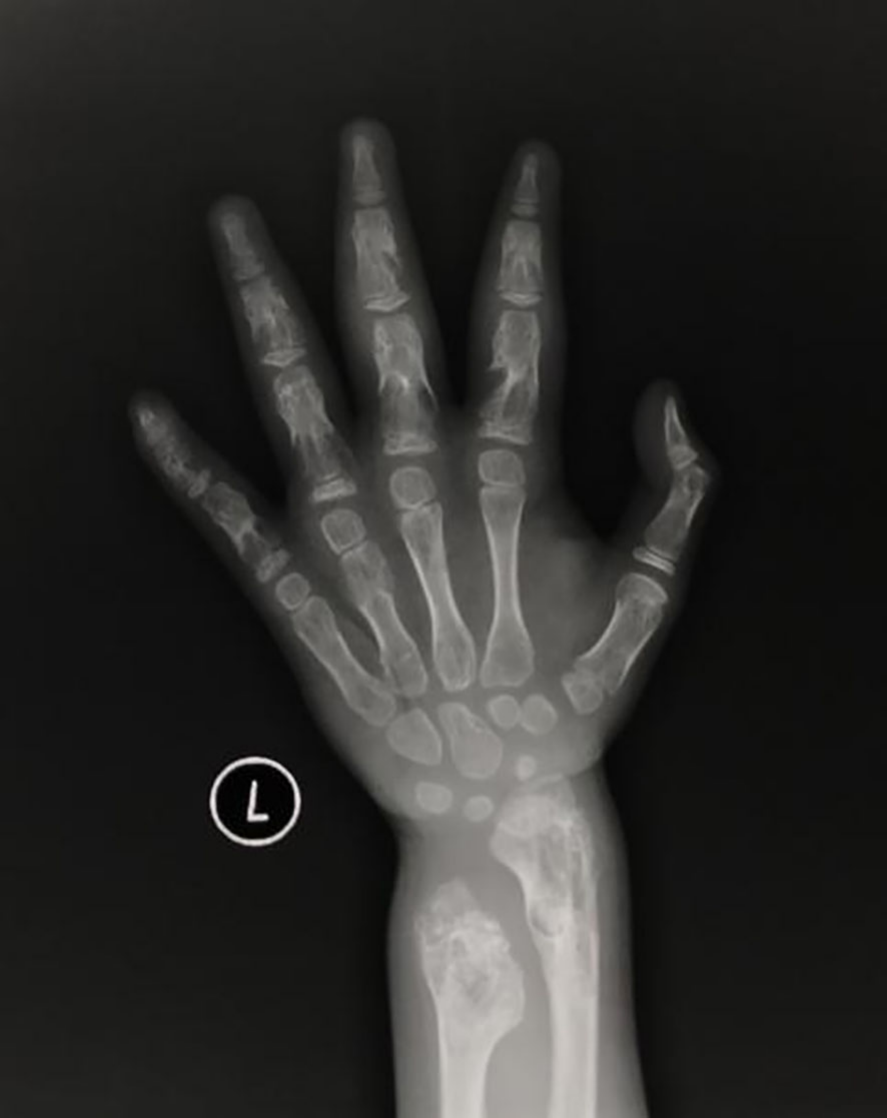
Plain bone X-ray showed multiple lytic bone lesions in left ulnar, radius and phalanges.

**Figure 3:**
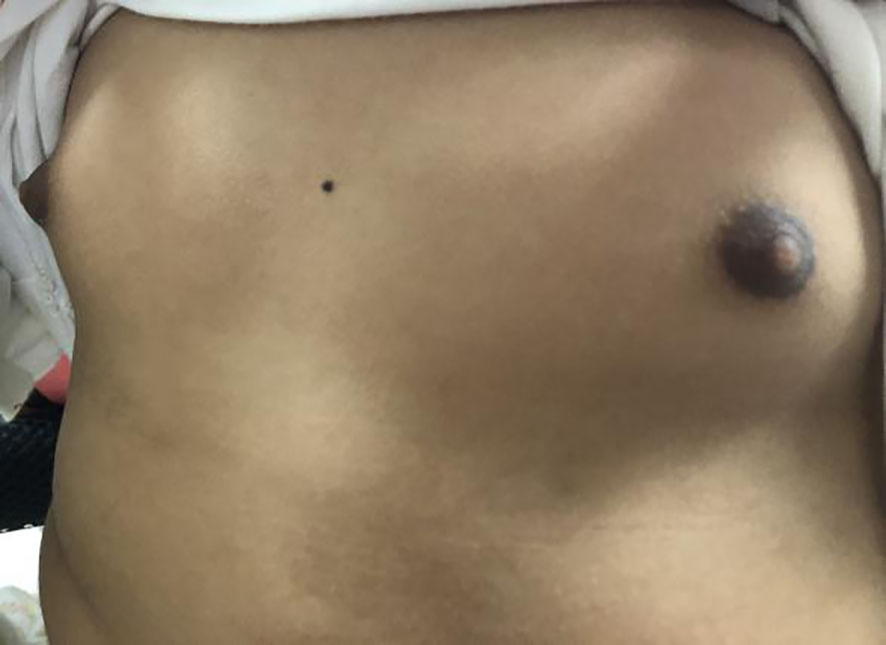
Bilateral breast development at Tanner stage III with chromatosis of the patient.

**Figure 4:**
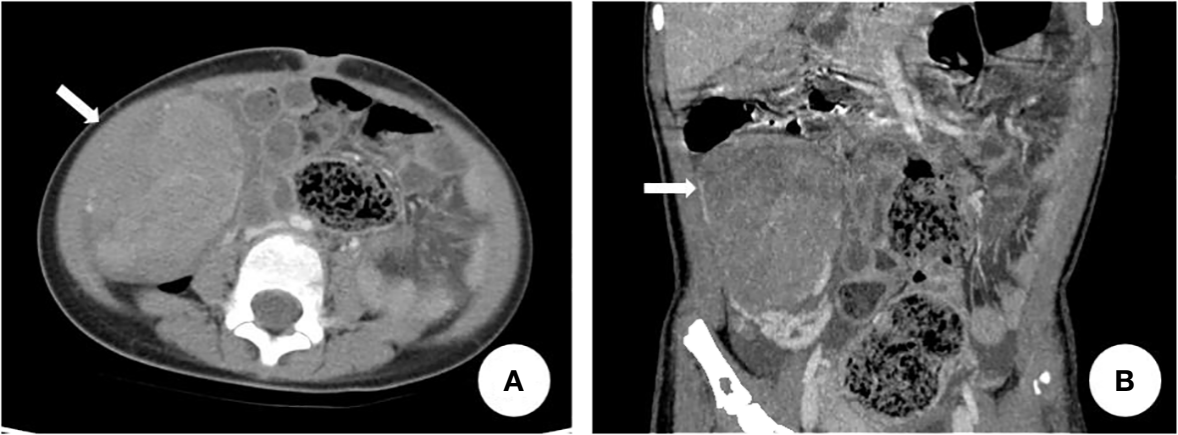
Abdominal CT revealed right lower abdominal soft tissue mass. The arrow in **(A)** and **(B)** indicated the right ovarian mass.

The patient was a 4-year-old female referred to the Department of Pediatric Orthopedics (Beijing Jishuitan Hospital, Beijing, China) with a primary diagnosis of OD. During physical examination of the patient, an asymmetrical involvement of the extremities caused by multiple enchondromas was discovered, especially right genu valgus deformity. Plain bone x-ray of the patient was requested for the limbs, and multiple lytic bone lesions were found in the distal part of the right femur, right tibia and fibula, left ulnar, radius, and phalanges (**Figures 1**, **2**). She underwent a wide resection of the soft tissue tumor in the distal right femur and artificial bone substitution. The tumor was sent for pathology test. Approximately 9 months later, the patient was referred to our clinic (Department of Pediatrics, Beijing Jishuitan Hospital) because of bilateral breast development. Physical examination showed bilateral breast development at Tanner stage III with chromatosis (**Figure 3**), hypertrophy of labia majora, and vulvar discharge. There was no other significant sign or symptom in her history or physical examination. The sex hormones suggested that estradiol (1,211 pmol/L, reference range: ≤ 115.6 pmol/L) and prolactin (43.99 ng/ml, reference range: 3.3–24.1 ng/ml) were elevated. The sonographer described a well-encapsulated multilocular–solid tumor, 81 mm ×76 mm ×57 mm, with intracystic fluid and moderate vascularity in the solid portion of the tumor originating from the right ovary. No invasion of the tumor in the adjacent organs was visible. Abdominal CT revealed a right ovarian solid mass (**Figure 4**). Right salpingo-oophorectomy was performed. The ovarian tumor was sent for pathology test. The study was approved by the Institutional Review Board of Beijing Jishuitan Hospital, and informed consent was signed by the guardian of the patient before enrollment.

### 2.2 Genetic test

Whole-exome sequencing and the Sanger method were used to detect gene mutation in the peripheral blood, ovarian tumor, and chondroma tissue. Genomic DNA was extracted from the peripheral blood samples of the patient and her parents. We carried out whole-exome sequencing on DNA using the IDT kit for a full exome probe to capture, enrich, and control the quality of the enriched library. Then, we used the high-throughput sequencer (Illumina) to sequence. Sequencing data were to be subject to strict quality control, and sequence comparison was to be conducted after it was confirmed to be qualified. After that, the GATK software was used for variable heterotopic point detection (SNP/dup/indel). Based on the self-developed software, Clinvar, ExAC, 1000 Genome, and other public databases, the HGMD commercial database and the self-built local database of the Chinese population, were called for gene function analysis. The American College of Medical Genetics (ACMG) standards and guidelines were applied to determine the pathogenicity of each variation (Beijing Huanuo Aomei Gene Biotech Co., Ltd.). The variation was divided into five levels: 1, benign; 2, likely benign; 3, variant of uncertain significance (VUS); 4, likely pathogenic; and 5, pathogenic. Later, Sanger sequencing with specific primers was conducted to confirm the identical variants of the proband and the parents. The frequency in the general population of the identified variants was checked using the single-nucleotide polymorphism (dbSNP) database and the 1000 Genomes Project. PolyPhen-2 and Mutation Taster were used to predict the possible protein functional changes caused by the variant. The American College of Medical Genetics (ACMG) standards and guidelines were applied to determine the pathogenicity of each variation (Beijing Huanuo Aomei Gene Biotech Co., Ltd.).

### 2.3 Validation of protein expression *in vitro*

#### 2.3.1 Generation of plasmids

The oligonucleotides of wild-type (WT) or mutant (Mut) NADP-dependent isocitrate dehydrogenase-1 (IDH1) CDS sequences were synthesized by IGEbio (Guangzhou, China). The WT or Mut sequences were cloned into the pcDNA3.1 expression plasmids.

#### 2.3.2 Cell transfection

HeLa cells were cultured in DMEM basic (1×) (Gibco, China, Cat. C11995500BT) supplemented with 10% fetal bovine serum (Biological Industries, Israel, Cat.04-001-1A) and 1% ampicillin–streptomycin (Hyclone, South Logan, Utah). Transfection was performed with the Lipofectamine™ 3000 Transfection Kit (Invitrogen, American, Cat. L3000-001) following the manufacturer’s instruction.

#### 2.3.3 Protein extraction and Western blotting analysis

For Western blotting, the cells were washed with PBS (Hyclone, South Logan, Utah) and lysed with RIPA Lysis Buffer (ThermoFisher, Cat. 89900) containing 1 mM PMSF (Sevicebio, Cat. G2008) for 5 min on ice. The samples were sonicated and centrifuged at 10,000 × *g* for 10 min at 4°C. The supernatant was collected, and a Pierce BCA Protein Assay (ThermoFisher, Cat.23227) was performed to determine the total protein concentration. The samples were diluted with 5× loading buffer (Fdbio Science, China, Cat. FD002) and heated at 98°C for 10 min. The protein samples were separated using 4%–12% Bis-Tris gels (GenScript, China, Cat. M00652) and a mini protein electrophoresis system (BIO-RAD, United States, Cat. 1658034) following the manufacturer’s instructions. The protein bands were then transferred to polyvinylidene fluoride (PVDF) membranes (Millipore, United States, Cat. IPVH00010) *via* a Mini Trans-Blot Electrophoretic Transfer Cell (Cat. 1703930, BIO-RAD, CA, United States). Next, the membranes were blocked with 5% bovine serum albumin (BSA) buffer and separately probed with rabbit anti-IDH1 (Proteintech, Cat. 12332-1-AP), rabbit anti-GAPDH (Servicebio, Cat. GB11002), rabbit anti-S6 (CST, Cat. #2217), and rabbit anti-Phospho-S6 (CST, Cat. #4856) overnight at 4°C with a final dilution of 1:1,000 (v/v). After washing three times, the membranes were incubated with HRP-conjugated goat anti-rabbit IgG (H+L) cross-adsorbed antibody (Invitrogen, United States, Cat. G-21234) at a 1:5,000 dilution (v/v), at 37°C for 1 h. The immunoreactive bands were detected and analyzed with a Bio-Rad ChemiDoc MP imaging System (BIO-RAD, United States Cat. 12003154) in conjunction with the Image Lab Software. The band intensities of the proteins of interest were quantified using ImageJ software.

### 2.4 Statistical analysis

Data were expressed as means ± standard deviation (SD) derived from at least three independent experiments. Student’s *t*-test was used to perform statistical analysis. *p*-value <0.05 was considered as statistically significant. *p < 0.05; **p < 0.01.

## 3 Results

### 3.1 Histopathological and immunohistochemical results

**Figure 5:**
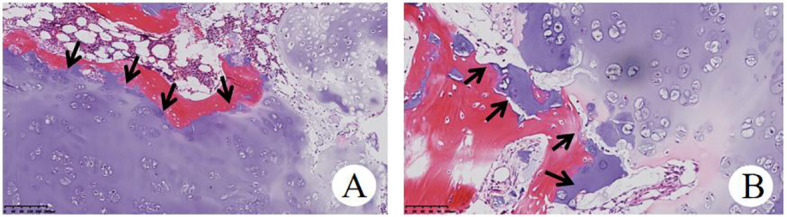
Histopathological examination of OD **(A)**, HE×100) and **(B)**, HE×200) showed that focal infiltration of cartilage tumor destroyed host bone.

**Figure 6:**
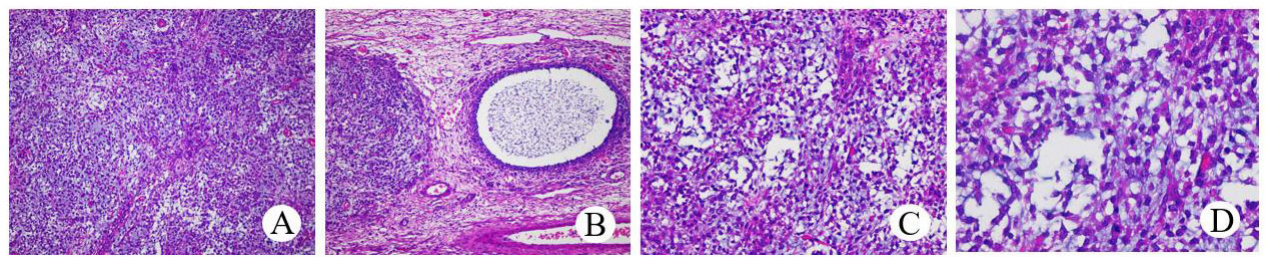
Histopathological examination of JGCT **(A)**, HE×100). The tumor cells were uniform in size and volume. The follicle **(B)**, HE×100) with a clear boundary and a circle or irregular shape was covered with one or more layers of granular cells, and the cavities contained alkalophilic liquid. Tumor cells **(C)**, HE×200; **(D)**, HE×400) in the solid area were diffusely arranged, and there were vesicle cells in the surrounding stroma.

The histological result of the soft tissue tumor confirmed the diagnosis of enchondromas (**Figure 5**). The histological report of the right ovarian solid mass revealed the diagnosis of a juvenile granulosa cell type (**Figure 6**). The patient’s JGCT was graded as International Federation of Gynecology and Obstetrics (FIGO) stage IA. The immunohistochemical result of the ovarian tumor was vimentin (+), AE1/AE3 (weakly, +), CK7 (−), EMA (−), CD117 (scattered +), ER (−), PR (+), CD99 (+), a-inhibin (single +), CD56 (+), CD30 (−), PLAP (−), SALL4 (−), OCT3/4 (−), HCG (−), Ki67 (approximately 30%, +), CK8 (−), SMA (+), desmin (−), and S-100 (−).

### 3.2 Genetic test results

**Figure 7:**
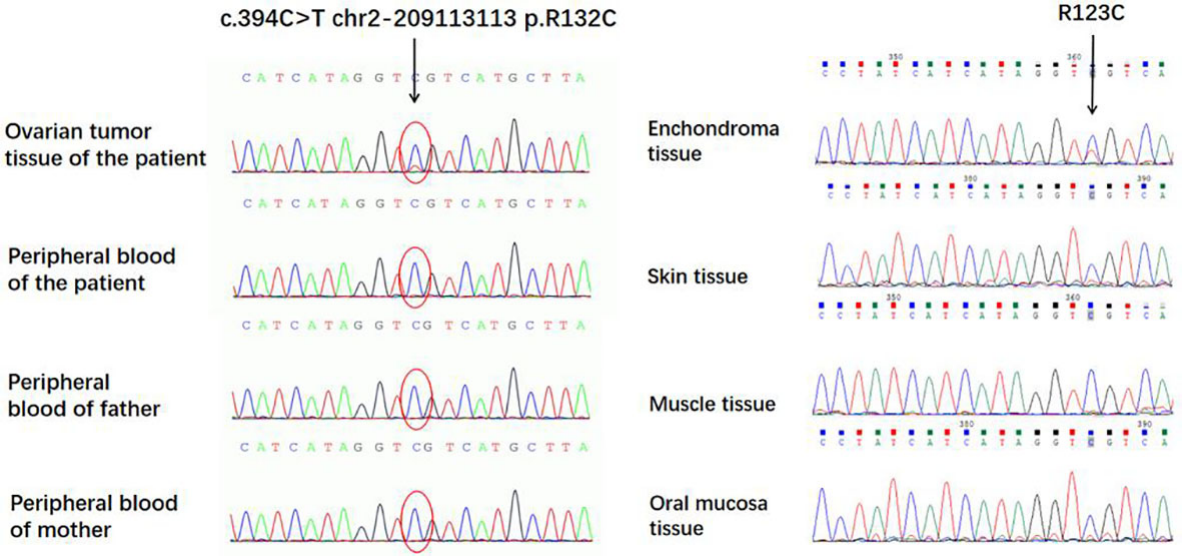
A c.394C>T (p.Arg132Cys) mutation of the IDH1 gene was detected in both the ovarian juvenile granulosa cell tumors and enchondroma but not detected in peripheral blood, skin tissue, muscle tissue, and oral mucosa samples of the patient.

A c.394C>T (p.Arg132Cys) mutation (**Table 1**) of the IDH1 gene was detected in both the ovarian JGCTs and enchondromatosis but not detected in the peripheral blood, skin tissue, muscle tissue, and oral mucosa samples of the patient, nor in the peripheral blood of the parents of the patient (**Figure 7**). The difference in mutated heterozygous peak height is generally due to the fact that the abundance of mutant cells in tumor tissues is less than 50%.

**Table 1:** The IDH1 mutation in ovarian juvenile granulosa cell tumor and enchondroma.

Gene	Genomic position	Transcript	Inheritance	Zygosity	Variant classification	ClinVar ID	Frequency in gnomAD population
IDH1	chr2-209113113	NM_005896.2	AD	Hemizygous	Ex4: c.394C>T, pArg132Cys (Pathogenic/Likely pathogenic​)	375891	0.00001

### 3.3 Experimental results *in vitro*

**Figure 8:**
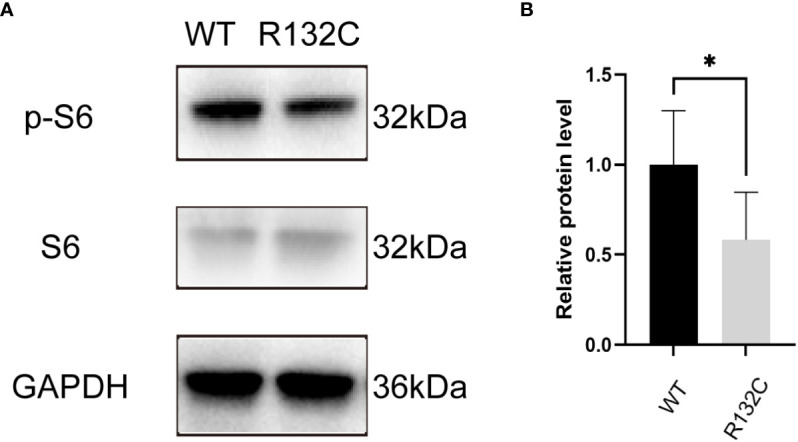
**(A)** Western blot analysis of p-S6, S6, and GAPDH proteins in cells transfected with IDH1 WT or Mut plasmid. **(B)** Quantitative analysis of the p-S6/S6 protein expression. *P<0.05 (Student’s t-test).

Transfection of HeLa cells with either WT or Mut plasmid caused 4.46- or 3.77-fold overexpression of the IDH1 gene compared to non-transfected control cells, respectively (**Supplemental Figure 1**). However, R132C mutation inhibited the phosphorylation of S6 ribosomal protein (S6), which is central to the mTOR pathway (**Figure 8**).

### 3.4 Follow-up results

Postoperatively, the estradiol (70.83 pmol/L, reference range: ≤115.6 pmol/L) and prolactin (20.54 ng/ml, reference range: 3.3–24.1 ng/ml) levels of the 4-year-old patient fell to values normal for her age in 1 week. When seen 3 months after the operation, there was no evidence of recurrence of either the multiple enchondromas or the ovarian tumor, and the remission of the pseudo-pubertal changes was complete. Estradiol and prolactin levels were normal until 2 years of follow-up.

## 4 Discussion

Here, we present 10 related cases reported through a literature search. Combined with our case, there were 11 cases. The clinical features, diagnosis, treatment, and prognosis of these cases are summarized in **Table 2**. In these 11 cases, the onset age ranged from 6 months to 36 years. There was no uniform rule between JGCT and OD. Both of them could occur on the same side of the limb, or OD could occur on one side of the limb, while JGCT occurred on the opposite side of OD. Pseudo-precocious puberty is the main clinical manifestation of ovarian JGCT patients before puberty, while abnormal menstruation, abdominal distension, and abdominal mass are the main clinical manifestations of ovarian JGCT for patients after puberty. As for the treatment of OD, tumors were surgically removed in eight cases, one case was conservatively treated, one case was amputated below the knee of the right leg, and one case involved osteotomy of right middle finger. As for the treatment of JGCT, eight cases underwent salpingo-oophorectomy on the diseased side and two cases received chemotherapy after operation. Except for two cases found with malignant transformation of chondrosarcoma, the remaining eight cases were benign lesions. Patients all had follow-ups starting from 6 months to 7 years post-operation, and no recurrences were noted.

**Table 2:** Review of JGCT associated with OD.

Author	Years	Age (years)		Symptoms		Treatment	Follow-up
		OD	JGCT	OD	JGCT		
Tamimi et al. (3)	1984	Infancy	15	Multiple skeletal deformities	Amenorrhea	RSO Bone tumor surgery	No recurrence (6 months)
Pounde et al. (4)	1985	16	15	Multiple rib masses	Abdominal mass	Right oophorectomy Chemotherapy Bone tumor surgery	Unknown
Vaz et al. (5)	1986	1.5	7	Legs and finger deformity	Sexual precocity	RSO Bone tumor surgery	Unknown
Velasco-Oses et al. (6)	1988	6	8	Multiple skeletal deformities	Sexual precocity	RSO Bone tumor surgery	No recurrence (7 years)
Asirvatham et al. (7)	1991	7	4	Left knee pain	Sexual precocity	Left oophorectomy Bone tumor surgery	No recurrence (5 years)
Tanaka et al. (8)	1992	11	15	Multiple skeletal deformities	Abdominal mass	LSO, Chemotherapy Bone tumor surgery	No recurrence (4 years)
Gell et al. (9)	1998	Unknown	13	Unknown	Abdominal discomfort, increased abdominal circumference	RSO Bone tumor surgery	No recurrence (1 year)
Rietveld et al. (10)	2009	Unknown	36	Unknown	No symptoms	LSO	No recurrence (10 months)
Burgetova et al. (11)	2017	22	0.5	Right leg pain	Pelvic pain	RSO Bone tumor surgery	No recurrence (4 years)
Jalaeefar et al. (12)	2019	Unknown	17	Hip pain	Abdominal distension, anorexia, and irregular menstruation	Right oophorectomy	Unknown
Current report	2021	3	4	Multiple skeletal deformities	Sexual precocity	RSO Bone tumor surgery	No recurrence (6 months)

OD, Ollier’s disease; GCT, granulosa cell tumor; LSO, left salpingo-oophorectomy; RSO, right salpingo-oophorectomy.

Ovarian JGCTs, first proposed by Scully (13) in 1977, make up 5% of ovarian granulosa cell tumors. Since granulosa cell tumors are nearly always hormonally (estrogen) active, preadolescent children may have precocious breast development, increased pubic hair, vaginal bleeding, anovulatory menstruation, or advanced growth and bone age, whereas older patients may have menstrual irregularities, abdominal pain, abdominal distension, and pelvic envelopes. Some patients have acute abdominal symptoms and signs because of rapid ovarian tumor growth or even torsion rupture, such as fever and ascites (14). In terms of immunohistochemistry, JGCT is positive for α-inhibin, calretinin, and SF-1, and it often expresses vimentin, WT-1, CD56, CD99, CD10, SMA, S100, and cytokeratin but rarely EMA (15). The production of estrogen by tumor cells manifests clinically as pseudoprecocious puberty in our patient; her ovarian mass tissue accords with the JGCT histological characteristics and immunohistochemistry. With the decrease of estrogen level after operation, her breast gradually retracted to the pre-puberty state, and the pigmentation of the areola gradually became shallow, which further supports JGCT of the ovary as the cause of pseudoprecocious puberty.

According to FIGO, the JGCT of our case is graded as stage IA, and the treatment is surgical resection of the right ovary and its appendages, without adjuvant radiotherapy and chemotherapy. Reviewing the related case reports before, most of them comprised the unilateral resection of the affected ovaries and appendages, and the overall prognosis was good. Because of the extremely low incidence of ovarian JGCT, various histological types, and complex biological behaviors, it is difficult to determine the best treatment plan. Therefore, a thorough staging must be performed, because this is the most valuable tool to predict outcomes and the extent of therapeutic measures needed at present. Besides laparotomy, staging evaluation should also include contralateral ovarian biopsy and peritoneal cytology. Surgery is still the main treatment. Most of the surgical experience of JGCT in children comes from case reports. Unilateral salpingo-oophorectomy is usually used to preserve reproductive function. Wedge biopsy and lymph node dissection of the contralateral ovary are not recommended, because sex cord–stromal tumors rarely have lymph node metastasis (16). Li et al. (17) suggest that surgical treatment is an important and effective method to treat pediatric ovarian tumors and improve the prognosis of children with malignant ovarian tumors. The application of radiotherapy and chemotherapy in JGCT is still unclear. Serum inhibin can be used as a specific marker of granular cell tumors and a reliable marker for detecting recurrence (14).

The clinical manifestation of OD in our case is multiple skeletal deformities of limbs. The x-ray film shows that multiple diseased bones are damaged, the expansion of bone cortex is interrupted, and the local density is uneven. The pathological tissue of bone tumors conforms to the manifestation of endogenous chondroma. Ollier’s disease often appears in early childhood, and its clinical manifestations are abnormal cartilage development in many parts and painless masses that can be touched by limbs. The pathogenesis is unknown, which is probably attributable to a failure of the normal enchondral ossification resulting from the proliferation of ectopic islands of chondroid tissue or to the incapacity of the epiphyseal plate to become mature, causing residual chondroid proliferation in the bones (18). The NADP-dependent isocitrate dehydrogenase (IDH1) gene mutations have been shown to be key events in the development of enchondromas (2). On x-ray film, enchondromas typically appear as osteolytic lesions (medullary) with well-defined, sclerotic margins, endosteal erosion, and ground glass appearance of the matrix, which is the main diagnosis of enchondromas. There are three main treatment methods: conservative treatment, simple curettage, and curettage bone grafting. In the absence of clinical symptoms, the treatment can be conservative. However, surgery is often needed when the growth is accelerated in a short time or local swelling or even pathological fracture occurs (19).

The correlated pathogenesis between OD and JGCT was not clear; previous scholars (9, 10) believed that it may originate from generalized mesodermal dysplasia. Early in embryonic development, intraembryonic mesoderm condenses into three contiguous structures adjacent to the notochord, the paraxial mesoderm, the intermediate mesoderm, and the lateral plate mesoderm. The lateral plate mesoderm ultimately forms the long bones of the skeleton, whereas the adjacent intermediate mesoderm forms the gonads. Therefore, the process disrupting the normal development of the long bones in OD may also affect the mesoderm that ultimately becomes the ovaries. In order to further explore the pathogenesis, whole-exome sequencing was carried out in the tissues of the JGCT of the ovary and peripheral blood of the patient, as well as the peripheral blood of the patient’s parents. To our knowledge, this is the first case in which gene detection was carried out among all reported cases of JGCT of the ovary complicated with OD. There was a somatic mutation in the IDH1 gene in JGCT of the ovary, the mutation site was c.394C>T (p. Arg132Cys), and this mutation was not carried in the peripheral blood samples of the patient and her parents. Sanger sequencing indicated that the IDH1 gene of multiple enchondromatosis tissue had the same mutation site as JGCT of the ovary, but this mutation was not detected in the skin tissue, muscle tissue, and oral mucosa of the patient, which has provided evidence for the role of somatic mutation in this patient and also further suggested that IDH1 somatic mutations may be the key factor in the pathogenesis of both tumors. IDH1 R132C and R132H mutations have been described in the tumors of 85% of patients with Ollier’s disease (20, 21). Heterozygous mutations in the IDH gene have been related to Ollier’s disease, mainly IDH1 (98%) and IDH2 (2%). These mutations exhibited a phenomenon of intraneoplastic mosaicism similar to that seen in fibrous dysplasia and osteochondroma (22). Kenny et al. (23) described a case of an ovarian cellular fibroma occurring in a patient with Ollier’s disease. They demonstrate an isocitrate dehydrogenase 1 (IDH1) mutation, which is characteristic of Ollier’s disease, within the ovarian neoplasm, suggesting a causal relationship. A previous study showed that IDH1 could affect cell migration by modulating the PI3K/AKT/mTOR pathway in primary glioblastoma cells (24). A previous study has further indicated that IDH^R132C^ mutation could regulate tumor growth by suppressing Akt signaling in intrahepatic cholangiocarcinoma (25). It is still unknown whether IDH^R132C^ mutation could regulate the mTOR pathway. In this study, we found that IDH^R132C^ mutation could suppress the phosphorylation of S6 ribosomal protein (S6) in HeLa cells. The regulatory mechanism remains to be further researched.

## 5 Conclusions

In conclusion, granulosa cell tumor of the ovary occurring with OD is rare. Surgery is still the main treatment for the two diseases. The IDH1 gene mutation may play a certain role in the pathogenesis of the two diseases, but it still needs to be further explored. Although most patients have a good prognosis in existing reports of JGCT with OD, the reported incidence of the malignant transformation of enchondromas in OD ranges from 5% to 50% (23). In children, periodic clinical screening every 6–12 months and plain radiographs of the known lesions every 2–3 years should be performed for early detection of growth abnormalities that need surgical treatment (26). Most cases of JGCT happen in the early stages, leading to good prognosis. However, it is necessary to closely follow the patients, as JGCT has an indolent course for possible recurrence, especially in the first or second decade (14). Therefore, regular postoperative follow-up of children is still essential to improve the survival rate.

## Data availability statement

The datasets presented in this article are not readily available because of privacy and ethical restrictions. Requests to access the datasets should be directed to zjdct@163.com.

## Ethics statement

The studies involving human participants were reviewed and approved by the Institutional Review Board of Beijing Jishuitan Hospital. Written informed consent to participate in this study was provided by the participants’ legal guardian/next of kin. Written informed consent was obtained from the individual(s) and minor(s)’ legal guardian/next of kin for the publication of any potentially identifiable images or data included in this article.

## Author contributions

JZ analyzed the patient’s data and participated in the writing and editing of the whole manuscript. RH participated in experiments *in vitro* and the writing of the manuscript. NW, LM, CL, YZ, XL, and TW participated in the conception of the study and literature review and assisted in the manuscript preparation. All authors contributed to the article and approved the submitted version.
