# Supplementary figures and images for: Ovarian juvenile granulosa cell tumors with Ollier’s disease in children with IDH1 gene somatic mutation
Source: Front Endocrinol (Lausanne). 2023 May 30;14:1093273. doi: 10.3389/fendo.2023.1093273 (PMC10265673; doi:10.3389/fendo.2023.1093273)

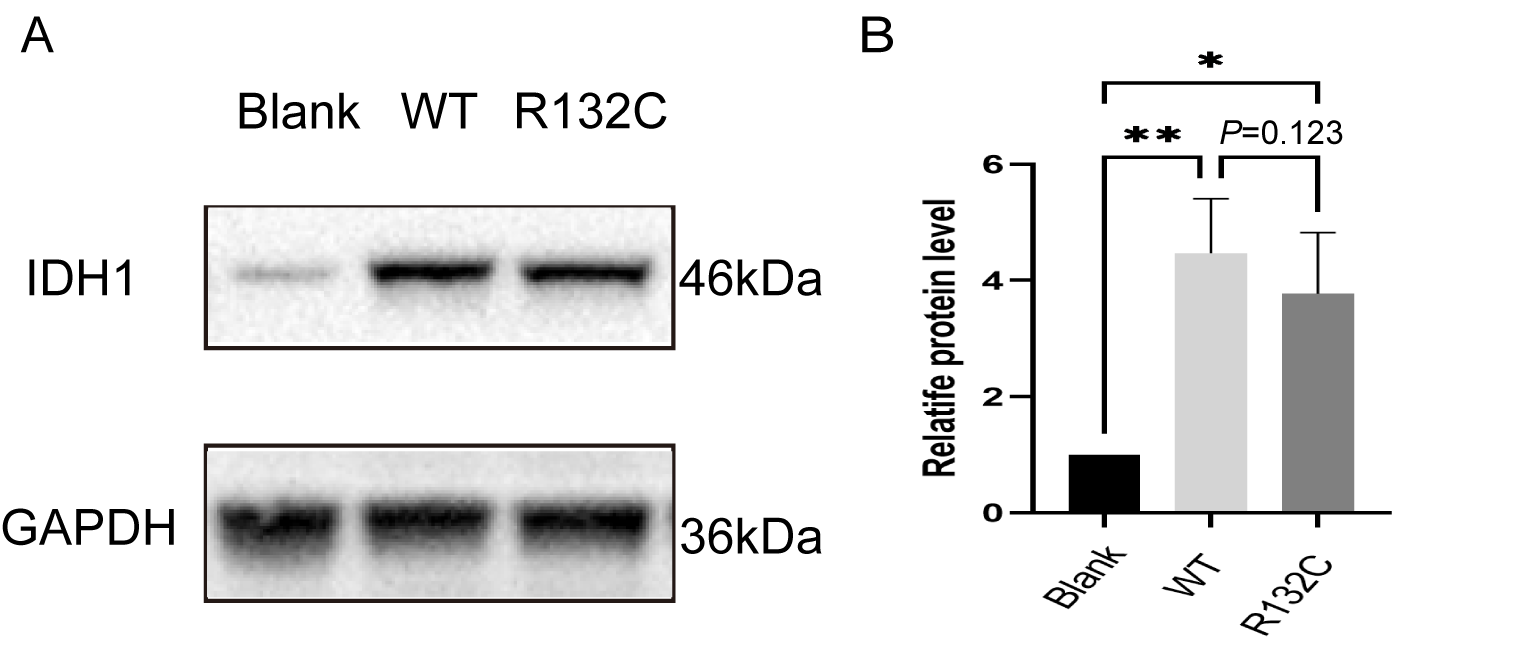

Supplement: Supplementary file 1 [file Image_1.tif]
